# Quantitative Analysis of 2D EXSY NMR Spectra of Strongly Coupled Spin Systems in Transmembrane Exchange

**DOI:** 10.1002/cbic.202300597

**Published:** 2023-12-04

**Authors:** Dmitry Shishmarev, Clement Q. Fontenelle, Bruno Linclau, Ilya Kuprov, Philip W. Kuchel

**Affiliations:** ^1^ The Australian National University John Curtin School of Medical Research 2601 Canberra ACT Australia; ^2^ The Australian National University Research School of Biology 2601 Canberra ACT Australia; ^3^ University of Southampton Department of Chemistry SO17 1BJ Southampton UK; ^4^ Department of Organic and Macromolecular Chemistry Ghent University Campus Sterre, Krijgslaan 281-S4 9000 Ghent Belgium; ^5^ The University of Sydney School of Life and Environmental Sciences 2006 Sydney NSW Australia

**Keywords:** exchange spectroscopy, GLUT-1, human erythrocyte, nuclear magnetic resonance, polyfluorinated sugar, *Spinach*

## Abstract

Solute translocation by membrane transport proteins is a vital biological process that can be tracked, on the sub‐second timescale, using nuclear magnetic resonance (NMR). Fluorinated substrate analogues facilitate such studies because of high sensitivity of ^19^F NMR and absence of background signals. Accurate extraction of translocation rate constants requires precise quantification of NMR signal intensities. This becomes complicated in the presence of *J*‐couplings, cross‐correlations, and nuclear Overhauser effects (NOE) that alter signal integrals through mechanisms unrelated to translocation. Geminal difluorinated motifs introduce strong and hard‐to‐quantify contributions from non‐exchange effects, the nuanced nature of which makes them hard to integrate into data analysis methodologies. With analytical expressions not being available, numerical least squares fitting of theoretical models to 2D spectra emerges as the preferred quantification approach. For large spin systems with simultaneous coherent evolution, cross‐relaxation, cross‐correlation, conformational exchange, and membrane translocation between compartments with different viscosities, the only available simulation framework is *Spinach*. In this study, we demonstrate GLUT‐1 dependent membrane transport of two model sugars featuring CF_2_ and CF_2_CF_2_ fluorination motifs, with precise determination of translocation rate constants enabled by numerical fitting of 2D EXSY spectra. For spin systems and kinetic networks of this complexity, this was not previously tractable.

## Graphical abstract

Quantitative interpretation of cross‐peaks in 2D EXSY spectra of compounds with strongly coupled spins undergoing transmembrane exchange required brute‐force numerical fitting to extract rate constants.

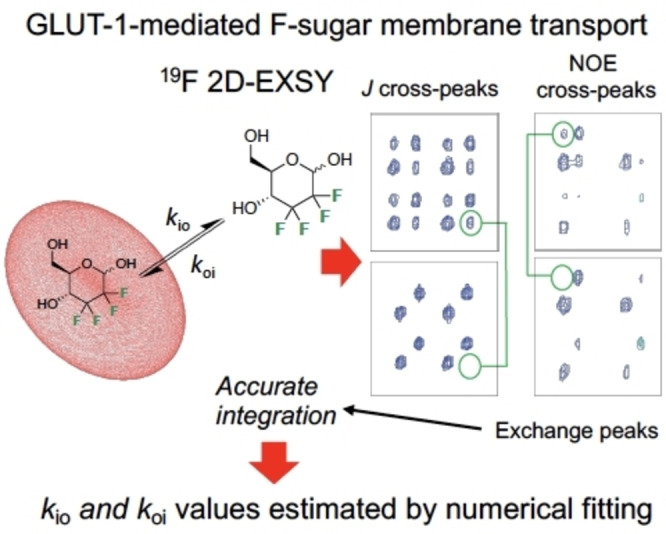

## Introduction

Exchange spectroscopy (EXSY) is a powerful nuclear magnetic resonance (NMR) technique that uses the fact that nuclear magnetisation travels with the nucleus during chemical reactions, conformational transitions, and physical processes.^[1]^ EXSY is routinely used for probing ion mobility in batteries,^[2]^ rearrangements in coordination complexes and organometallic compounds,^[3]^ stereochemical transitions,^[4]^ dynamics of supramolecular complexes,^[5]^ lateral diffusion in biological membranes,^[6]^ and transmembrane exchange.^[7]^

The two‐dimensional (2D) variant of EXSY can identify specific sources and destinations of magnetisation transfer through distinct 2D cross‐peaks. Although it is both selective and quantitative,^[8]^ accurate EXSY data processing requires rigorous quantification of NMR signals. Once this is achieved, well‐established data analysis procedures can be applied^[9]^ to both two‐site and multi‐site exchanges.[^8^, ^10^]

Accurate quantification of EXSY spectra can be confounded by processes that, although physically distinct, generate algebraically similar terms in the equation of motion, and thus complicate 2D EXSY spectra. One such process is the nuclear Overhauser effect (NOE), which moves magnetisation between nearby spins^[11]^ and consequently competes with chemical exchange. While specific mitigations exist for certain instances, such as exchange involving the solvent,^[12]^ separating NOE and exchange processes remains difficult. Another source of complications is homonuclear *J*‐coupling, which can alter cross‐peak intensity and introduce spurious cross‐peaks even in the absence of exchange.^[13]^ Some of these artefacts (from single‐quantum and double‐quantum pathways) can be suppressed by phase cycling,^[13a]^ while others (from zero‐quantum pathways) are more resilient and require complex NMR experiments employing pulsed field gradients.^[14]^ New complications, such as scalar cross‐relaxation,^[15]^ partner spin flip cross‐peaks,^[16]^ and geminal fluorine coupling effects,^[17]^ continue to be discovered.

**Figure 1:**
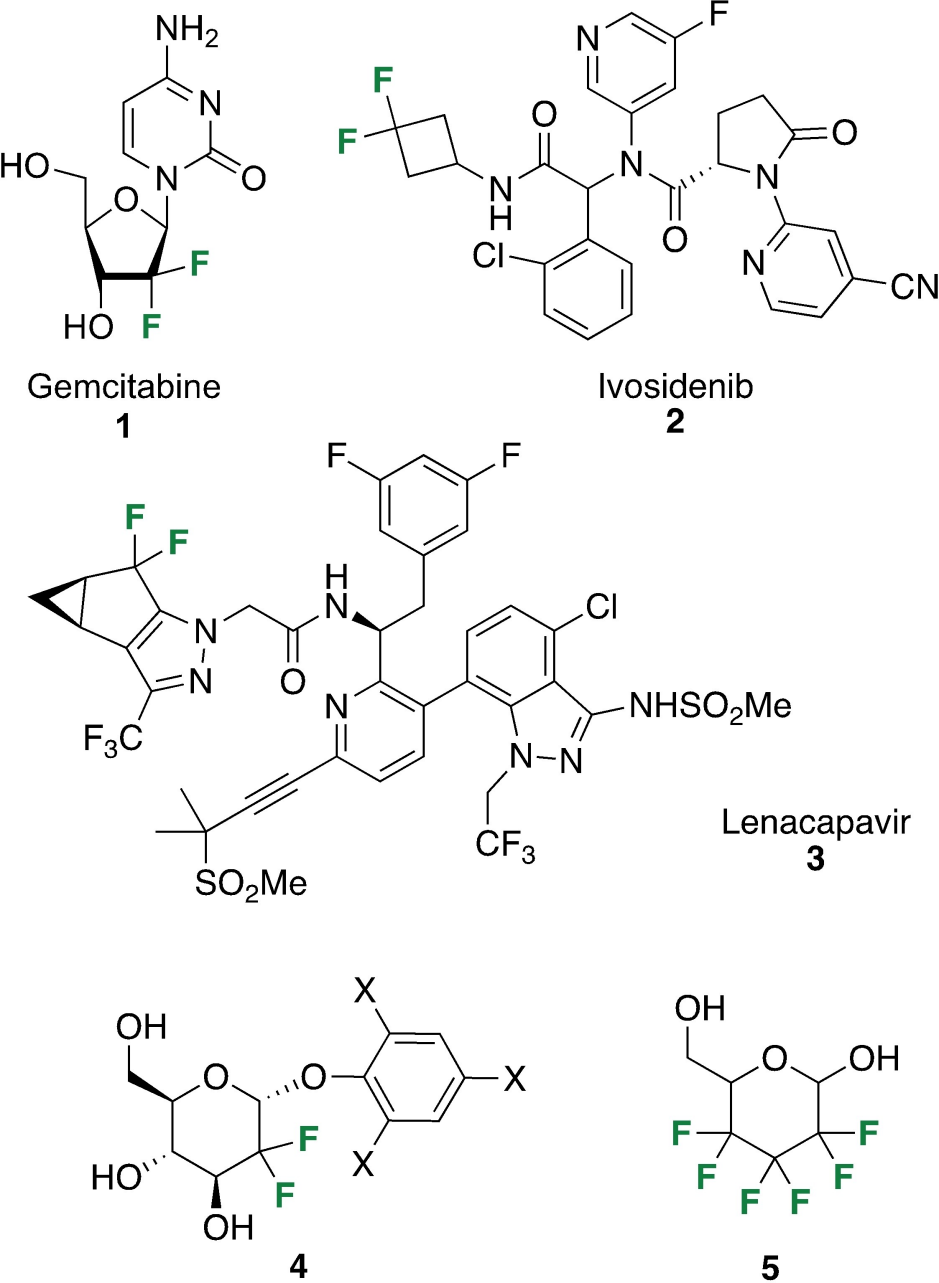
Examples of drugs and probes that contain geminal difluoro (−CF_2_−) group: gemcitabine (**1**), ivosidenib (**2**), lenacapavir (**3**), a difluorinated glucose probe used in the study of glycosidase enzymes (**4**), a hexafluorinated glucose probe used to investigate the ‘polar hydrophobicity’ phenomenon (**5**).

Accurate quantification of ^19^F 2D EXSY NMR spectra has become important due to the growing use of fluorinated drugs in the life sciences and pharmacology, as well as expansion into cell imaging and diagnostics.^[18]^ The geminal difluoro group (CF_2_) is common in many drugs, including the established anti‐cancer drug gemcitabine (**1**, Figure 1).^[19]^ Examples of CF_2_‐containing active components of recently approved drugs include ivosidenib **2** (leukemia)^[20]^ and lenacapavir **3** (multiresistant HIV).^[21]^ Many probes used in chemical biology also contain CF_2_‐groups, for example the difluorinated glucose derivative **4**, which has been used in glucosidase enzyme mechanistic studies.^[22]^ One particularly interesting compound is the hexafluorinated glucose **5**, featuring a −(CF_2_)_3_− chain. It was used in combination with 2D EXSY to investigate the ‘polar hydrophobicity’ phenomenon on the kinetics of the GLUT‐1 transporter protein in human red blood cells (RBCs).^[23]^

In protein‐carbohydrate interaction research, ^19^F NMR is used for carbohydrate epitope mapping, facilitating the identification of binding interactions,^[24]^ and carbohydrate fluorination is used in mechanism inhibitor design^[25]^ and is one of the possible avenues for glycomimetic drug development.^[26]^ Structural optimisation of carbohydrates has produced many glycomimetic drugs, such as oseltamivir (Tamiflu).^[27]^

**Figure 2:**
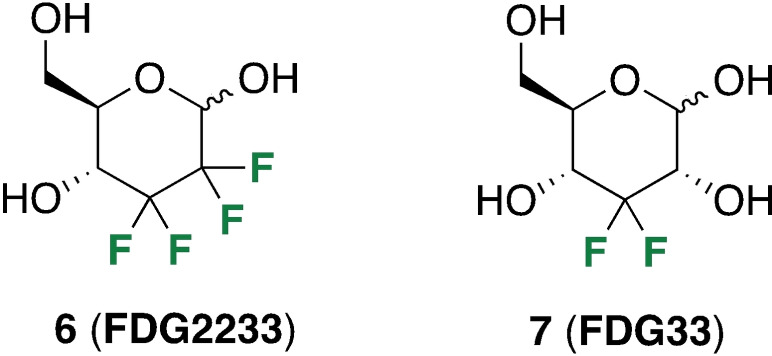
Specific CF_2_‐containing substrates employed in this study for investigating erythrocyte membrane transport using 2D EXSY NMR spectroscopy.

Following the earlier work on RBC membrane transport of hexafluorinated **5** ^[23]^ and 2,3,4‐trideoxy‐2,3,4‐trifluorinated glucose,^[28]^ we had set out to investigate membrane transport of glucose substrates with different fluorination patterns using EXSY NMR; the compounds included 2,3‐dideoxy‐2,2,3,3‐tetrafluorinated glucose (**6**, Figure 2; FDG2233)^[29]^ and 3‐deoxy‐3,3‐difluorinated analogue **7** (FDG33).^[30]^

We found that the established back‐transformation analysis methodology for ^19^F 2D EXSY quantification (cross‐peak identification, integration, and kinetic model fitting) of polyfluorinated substrates was no longer tractable due to additional quantum mechanical spin processes (listed in the Introduction) that do not exist in monofluorinated substrates. To overcome this obstacle, we used numerical least squares fitting of the two‐dimensional experimental spectra using *Spinach* software that explicitly models all aspects of coherent, dissipative, and chemical spin dynamics, including a pulse‐by‐pulse implementation of the 2D EXSY sequence in the time domain.^[31]^ By fitting the simulated 2D EXSY spectra directly to the experimental data, assuming DFT energy minimum geometries (detailed in the Supplementary Information), we extracted spin system parameters, rotational correlation times, and kinetic rate constants. This approach only recently became possible due to the availability of computers equipped with 128+ CPU cores, FP64‐capable GPUs, matrix‐based programming languages such as *Matlab*, and recent polynomial complexity simulation methods for liquid state NMR experiments.[^31a^, ^31c^, ^32^]

## Results and Discussion

**Figure 3:**
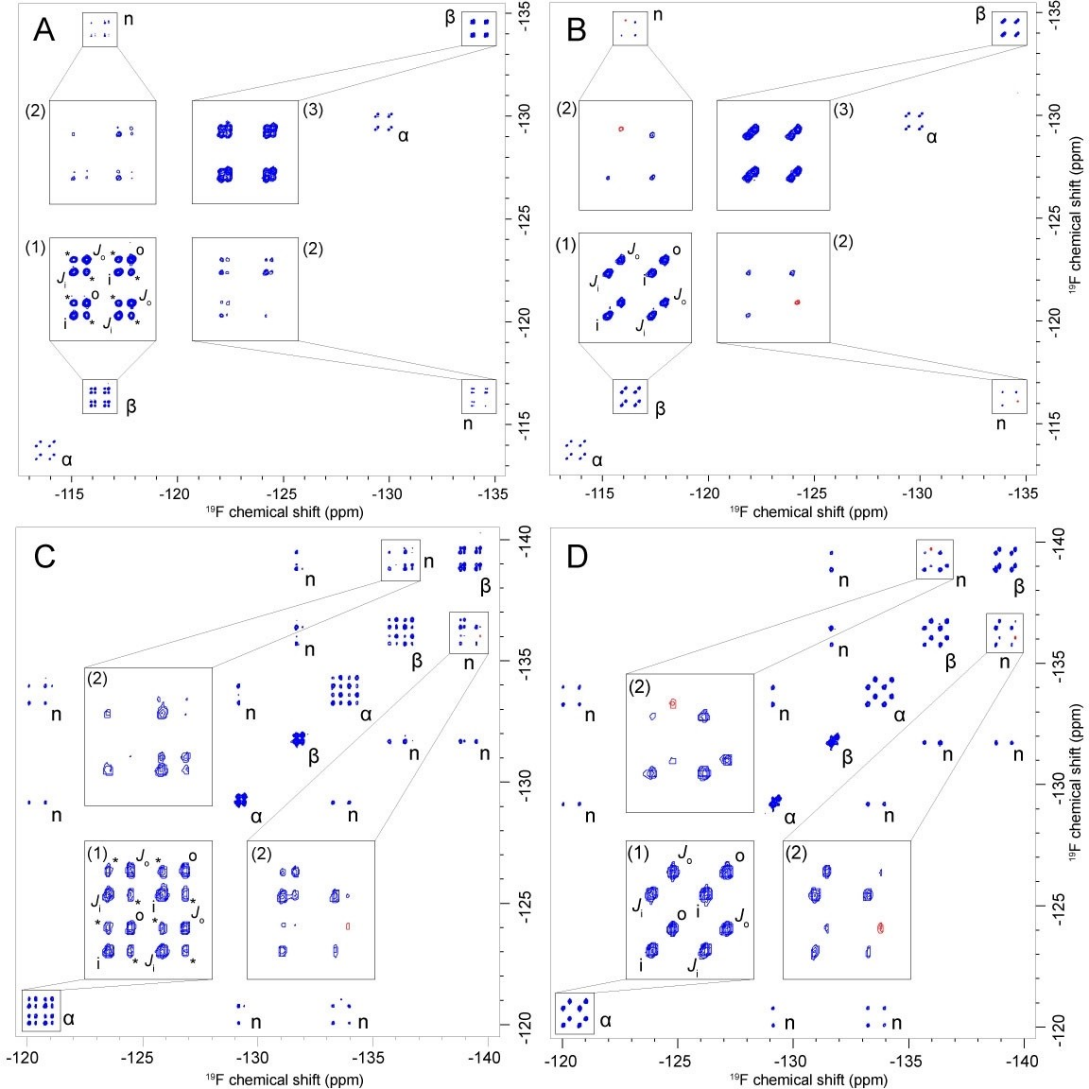
(**A**) ^19^F (376.46 MHz) 2D‐EXSY NMR spectrum of 20 mM FDG33 (**7**) in an RBC suspension, recorded with a mixing time of 500 ms at 37 °C. (**B**) Same as (A), in the presence of 0.1 mM cytochalasin B, an inhibitor of the GLUT‐1 glucose transporter. (**C**) ^19^F (376.46 MHz) 2D‐EXSY NMR spectrum of 20 mM FDG2233 (**6**) in an RBC suspension, recorded with a mixing time of 500 ms at 37 °C. (**D**) Same as (**C**), in the presence of 0.1 mM cytochalasin B, an inhibitor of the GLUT‐1 glucose transporter. Anomeric assignments are indicated by ‘α’ and ‘β’. Blue colour represents positive contour levels, whereas red denotes negative levels. Insets emphasise the intricacy of the observed peak groups. Signals originating from inside and outside of the RBCs are marked with ‘i’ and ‘o’, respectively. Peaks arising from ^19^F−^19^F J‐coupling are denoted by ‘*J*_i_’ and ‘*J*_o_’, while transmembrane exchange peaks are highlighted by asterisks, which disappear upon the introduction of cytochalasin B. Peak groups arising from NOEs are denoted by ‘n’. A few negative NOE cross‐peaks proceed from a combination of shorter rotational correlation time on the outside of the RBC and strong homonuclear J‐coupling; the same peaks are seen in simulations. The choice of the mixing time is dictated by the time scale of kinetics and cross relaxation, as well as by cell viability constraints on the experiment duration.

The work with FDG33 and FDG2233 started by establishing whether membrane transport could be observed, and if such transport was via the GLUT‐1 transporter. The ^19^F NMR 2D‐EXSY spectra of FDG33 and FDG2233 in an RBC suspension are shown in Figures 3A and 3C, respectively. As observed in the case of monofluorinated sugars,^[33]^ both FDG33 and FDG2233 exhibited distinct NMR peaks for species inside (‘i’) and outside (‘o’) the cells (Figures 3A–D(1)). Additionally, the presence of exchange peaks, marked with asterisks, confirmed the occurrence of chemical exchange across the membranes. The spectra in Figures 3B and 3D were recorded in the presence of cytochalasin B, a known GLUT‐1 inhibitor.^[34]^ The disappearance of the exchange peaks upon the inhibitor's introduction (compare Figures 3A(1) with 3B(1), and 3C(1) with 3D(1)) confirmed that the transmembrane exchange of FDG33 and FDG2233 was mediated by the GLUT‐1 transporter in RBCs, as opposed to passive diffusion. Due to the large ^2^ *J*_F−F_‐couplings between geminal ^19^F nuclei, the doublet components were clearly resolved, enabling us to identify a rare type of cross‐peaks between the two components of each doublet (peaks labelled ‘*J*_i_’ and ‘*J*_o_’ in Figures 3A–D(1)). These cross‐peaks, distinct from those described by Macura *et al*.,[^13a^, ^13b^, ^13c^] originate from longitudinal relaxation of the fluorine partner spin during the mixing time, similar to that observed in heteronuclear systems.^[16]^ In addition, other cross‐peaks were observed (Figure 3A–D(2)), which could be shown, by inhibition studies, to contain exchange contributions as well (compare Figures 3A(2) with Figure 3B(2), and 3C(2) with 3D(2)). Hence, the high spectral resolution of our 2D EXSY spectra allowed us to identify intricate patterns of combined *J*‐coupling and NOE cross‐peaks with exchange peaks, which could have been overlooked at insufficient resolution, but which need to be accounted for to achieve quantitative integration of the exchange peaks.

**Figure 4:**
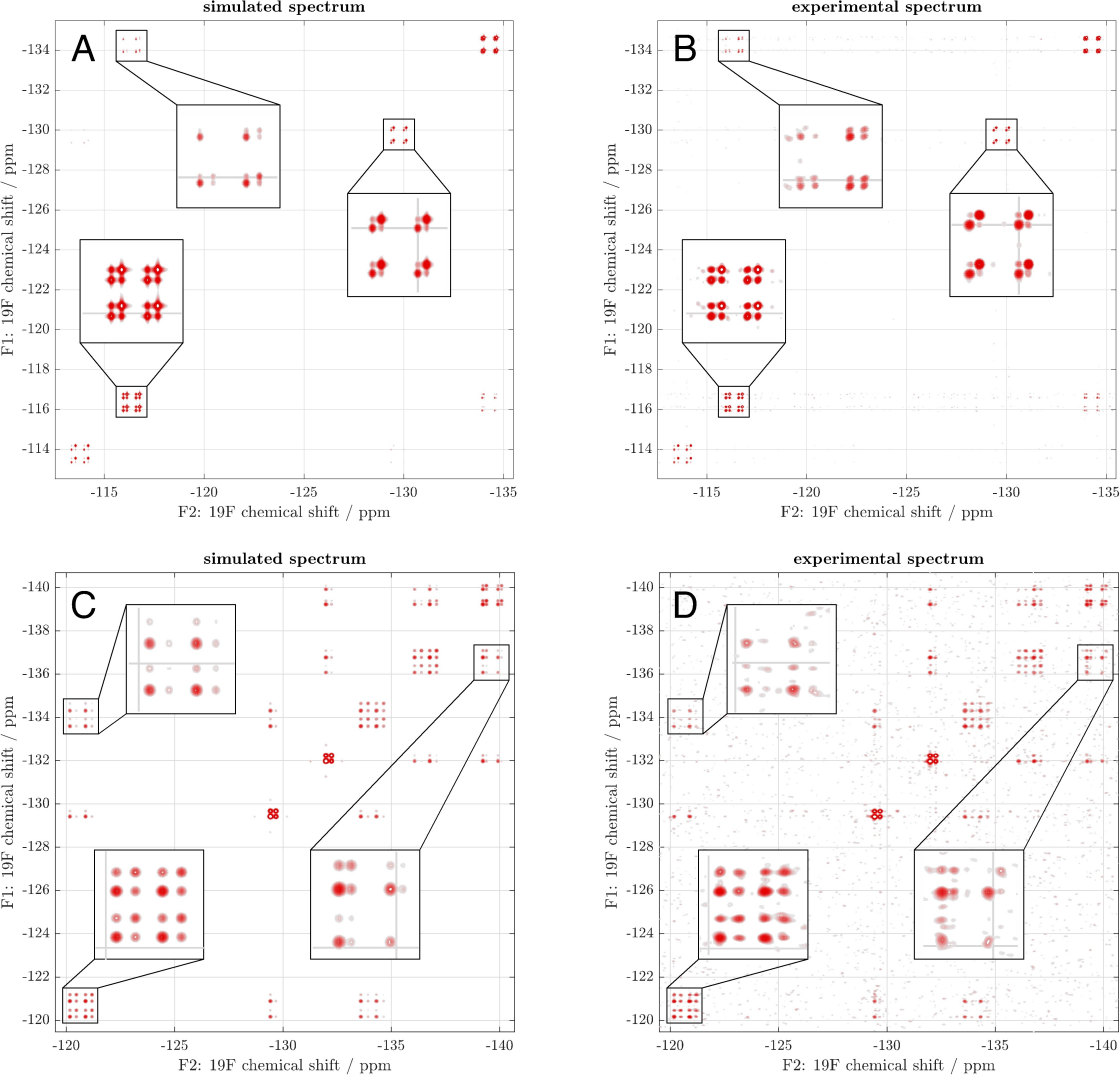
Comparative view of simulated and fitted ^19^F 2D‐EXSY NMR spectra (panels A and C) against corresponding experimental spectra (panels B and D) of FDG33 (panels A and B) and FDG2233 (panels C and D). Deviation histograms between the fit and the experimental data are both Gaussian with a zero mean (see Figure S1 in the Supplementary Information). The fitting is done using a numerical least squares procedure that minimises the squared Frobenius norm of the difference between 2D spectra with respect to the adjustable parameters given in Tables 1 and S1.

However, the processes arising from ^19^F−^19^F *J*‐couplings and NOEs are not accounted for by the traditional two‐ or multi‐site exchange theory that is used in conventional EXSY quantification methods. In addition, the disappearance of some peaks within the spectral groups on adding cytochalasin B (e. g. Figures 3B(2) and 3D(2)) suggested that some of the peaks were a consequence of transmembrane exchange. In view of this superimposed complexity, we were compelled to adopt a ‘brute‐force’ numerical approach, using least squares fits of two‐dimensional spectra to extract the translocation rate constants. The 2D EXSY spectra shown in Figure 3 contain sufficient information to constrain all relevant parameters: chemical shifts, *J*‐couplings, concentrations of α‐ and β‐glucose (anomerisation is too slow to be observed in this context), apparent rate constants for influx and efflux (which differed for α‐ and β‐anomers), and rotational correlation times. The aqueous suspension medium for RBCs and the haemoglobin‐packed cytoplasm (~340 g L^−1^) are known to have significantly different bulk‐ and micro‐viscosities, which would suggest different rotational correlation times,^[35]^ something that the theory team had independently found to be necessary to obtain a good fit. Although finding a suitable initial guess in the large parameter space (34 parameters for FDG2233 and 19 parameters for FDG33) was laborious, the resulting fits, as shown in Figure 4, were unambiguous and precise (as inferred from the difference histograms shown in the Supporting Information). The source code for the simulations and fitting are given in the example set of *Spinach* 2.8 and later versions.

Figure 4 compares the fitted (left panels) and the experimental (right panels) ^19^F EXSY spectra of FDG33 (top row) and FDG2233 (bottom row). The fitted parameters, a selection of which is shown in Table 1 (the complete list is in Table S1), provide insights into the transmembrane exchange kinetics of these fluorosugars. We were/are particularly interested in the apparent rate constants for efflux (*k*_io_) that are, unlike apparent rate constants for influx (*k*_oi_), independent of the haematocrit of the sample. Notably, the *k*_io_ values showed anomer‐specific differences for both FDG33 and FDG2233. The rate constants were lower for the α‐ than the β‐anomer for FDG33, whereas they were slightly higher for the α‐anomer for FDG2233. These findings indicate a preference of GLUT‐1 for certain anomers, particularly in the case of FDG33; they reinforce existing knowledge for monofluorinated glucose analogues.^[33]^ Additionally, the tetrafluorinated sugar has greater exchange rate constants than its difluorinated counterpart.

**Table 1:** Key fitted parameters used in the simulations shown in Figure 4.

Substance	Parameter		Anomer	
			α	β
FDG33 **7**	*k*_io_ (s^−1^)^[a]^		0.23±0.03	0.79±0.03
	*k*_io_ (s^−1^)^[b]^		0.23	0.84
	*τ*_c_ (s)^[a]^	inside	10^−9.03±0.01^	
		outside	10^−9.28±0.01^	
FDG2233 **6**	*k*_io_ (s^−1^)^[a]^		0.94±0.05	0.87±0.04
	*k*_io_ (s^−1^)^[b]^		1.07	0.83
	*τ*_c_ (s)^[a]^	inside	10^−8.46±0.01^	
		outside	10^−9.09±0.02^	

[a] Two‐dimensional fitting using *Spinach*. [b] Back‐transformation.

To compare numerical fitting with the traditional EXSY analysis method, we calculated the apparent efflux rate constants using the back‐transformation method, which did not account for NOE or *J*‐coupling cross‐peaks. We uncovered a deviation from the values obtained using *Spinach*, which considers all quantum‐mechanical pathways affecting the experiment, including NOE. For instance, the α‐anomer of FDG2233 exhibited a rate constant of 1.07 s^−1^ via back‐transformation, compared to 0.94±0.05 s^−1^ obtained with *Spinach*. A similar disparity was noted for the β‐anomers of FDG2233 and FDG33 (Table 1).

The rotational correlation times estimated for both FDG33 and FDG2233 showed significant differences between the interior and exterior of the RBCs (Table 1). This highlights the importance of accounting for factors such as different extents of molecular crowding and the different viscosity of the intra‐ and extracellular spaces when studying transmembrane exchange processes. These factors could potentially influence how the fluorosugars interact with the GLUT‐1 transporter on either face of the plasma membrane, subsequently affecting the overall transport kinetics.

Some rotational correlation times are positioned near the zero crossing of the homonuclear Overhauser effect rate curve. This is not a problem: a near‐zero cross‐peak intensity is still a valid mathematical constraint during fitting. The “outside” correlation times in Table 1 are similar (and reproducible) because cells were washed with the same suspension medium every time. However, the “inside” correlation times are both very different between FDG33 and FDG2233, and the estimates were not reproducible; this was likely to be due to the two species binding differently to various macromolecules inside the cells, and under subtly different metabolic conditions.

## Conclusions

We demonstrate that a 3,3‐difluorinated, and a 2,2,3,3‐tetrafluorinated glucose derivatives are both reversibly transported through the erythrocyte membrane via GLUT‐1, and that a variety of quantum mechanical complications in EXSY spectroscopy (cross‐correlated relaxation,^[36]^ NOESY cross‐peaks,^[11]^ strong *J*‐coupling artefacts,[^13a^, ^13b^, ^13c^] partner spin flip cross‐peaks,^[16]^ scalar cross‐relaxation,^[15]^ and potentially others yet to be discovered) may be circumvented, and even put to good use, by performing direct fitting of a detailed simulation to the 2D spectrum in order to extract estimates of the transmembrane exchange rate constants. This was previously infeasible for systems of this complexity; this new functionality of the Spinach library is applicable to any other type of NMR spectrum. Estimating exchange rate constants with high precision is essential for advancing the understanding of membrane transport kinetics and creating novel strategies to study these processes *in vivo*. Accurate quantification of 2D‐EXSY spectra enables deeper insights into the dynamics of polyfluorinated compounds, sheds further light on their interactions within biological systems, and facilitates the development of new therapeutic agents, cellular imaging techniques, and diagnostic methods, ultimately enabling broader understanding of reactions in heterogeneous biological environments.

## Experimental Section

**Fluorinated sugar derivatives**. The tetrafluorinated sugar derivative **6** was obtained using a synthetic route developed in our laboratory.^[29]^ The difluorinated glucose analogue **7** was produced by adapting established methods.[^30^, ^37^] Stock solutions of fluorosugars were prepared in deuterated saline that consisted of 154 mM NaCl in 99 % D_2_O sourced from the Australian Institute of Nuclear Science and Engineering (Lucas Heights, NSW, Australia).

**RBC sample preparation**. Fresh blood was obtained from a single consenting donor, following approval from the University of Sydney Human Ethics Committee. The venipuncture was via the cubital fossa, and the volume of each sample was ~20 mL. The samples were anti‐coagulated with 15 IU (mL blood)^−1^ of heparin and centrifuged at 3000 *g* for 5 min at 10 °C. This process enabled the isolation of RBCs via vacuum‐pump aspiration of blood plasma and the buffy coat.

The RBCs were then washed twice in saline (154 mM in Milli‐Q H_2_O) and then in “inosine saline” (composed of 10 mM inosine, 10 mM sodium pyruvate, 5 mM NaH_2_PO_4_, 4.5 mM NaOH, 10 mM KCl, and 133 mM NaCl in Milli‐Q H_2_O, pH 7.4). The washing process involved the re‐suspension of cells in ~5 volumes of the washing medium, centrifuging at 3000 *g* for 5 min at 10 °C and removing the supernatant by vacuum‐pump aspiration. Prior to the final wash, cells were gently bubbled with carbon monoxide for 10 min, which converted the haemoglobin into its stable diamagnetic form, resulting in improved NMR resolution and signal‐to‐noise ratio.

For NMR experiments, RBC suspensions were mixed with stock solutions of fluorosugars in 5‐mm NMR tubes. In all ^19^F NMR experiments, the total sample volume was 0.5 mL, containing 10 % (v/v) D_2_O for locking the magnet field; and haematocrits were adjusted to be between 60 % and 70 %. Cytochalasin B, purchased from Sapphire Bioscience (Redfern, NSW, Australia), had a 20 mM stock solution prepared in dimethyl sulfoxide‐d_6_. For GLUT‐1 inhibition studies, typically 2.5 μL of this solution was added to a 0.5 mL NMR sample, yielding a final concentration of 0.1 mM. All other reagents were sourced from Sigma‐Aldrich (St Louis, MO, USA).

**NMR spectroscopy**. NMR spectra were recorded using a Bruker Avance III 400 spectrometer (Bruker BioSpin, Karlsruhe, Germany), equipped with a 5‐mm dual ^19^F/^1^H probe, operating at a sample temperature of 37 °C. Bruker TopSpin 3.5 software was used for instrument control and processing of the NMR spectra.

We performed 2D EXSY experiments with FDG33 and FDG2233 following the methodology described in our previous work on monofluorinated sugars.^[33]^ Specifically, WALTZ‐16 composite‐pulse decoupling (90° decoupler‐pulse duration of 90 μs centred at 4 ppm) and a high‐power 180° ^1^H pulse were used for proton decoupling during the direct and indirect acquisition periods, respectively. Axial peaks were suppressed by a two‐step phase cycle for the first 90° pulse. We used an inter‐transient delay of 8 s and a mixing time of 500 ms.

The acquired FID matrix consisted of 1024 complex points in the direct acquisition dimension, and 512 complex points in the indirect acquisition dimension. Data processing involved the application of a cosine‐squared window function in both dimensions. The indirect acquisition dimension was extended to 1024 complex points using the in‐built linear prediction algorithm in TopSpin. For back‐transformation analysis, the volumes of two‐dimensional peaks were measured using peak integration in TopSpin. For further analysis in *Spinach*, the spectra were exported into *Matlab* as ASCII text files.

**NMR simulations**. Simulations were carried out using *Spinach* library^[31a]^ version 2.8, which explicitly solves the Liouville – von Neumann equation of motion for the EXSY pulse sequence in the time domain and in Liouville space.^[32]^ For a spin system undergoing coherent evolution, relaxation, and membrane translocation, the magnetokinetic equation of motion, discussed in detail in,^[38]^ is automatically generated and solved by *Spinach*:

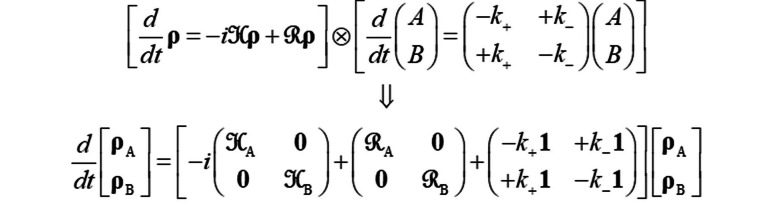




where 
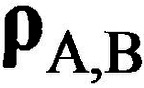
 are density matrices of the endpoints of the translocation process, 
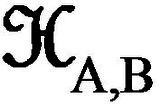
 are their isotropic Hamiltonian commutation superoperators, 
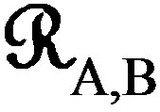
 are their relaxation superoperators, and 
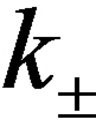
 are forward and reverse translocation rate constants.^[7]^ All relevant complications (cross‐correlated relaxation,^[36]^ NOESY cross‐peaks,^[11]^ strong *J*‐coupling artefacts,[^13a^, ^13b^, ^13c^] partner spin flip cross‐peaks,^[16]^ scalar cross‐relaxation,^[15]^ *etc*.) are accounted for automatically in the relaxation superoperator.

The isotropic part of the spin Hamiltonian included chemical shift and *J*‐coupling terms. They were treated as adjustable parameters during simplex least‐squares fitting (Matlab's fminsearch function) to experimental 2D EXSY spectra. Initial guess values were obtained from density functional theory (DFT) calculations, and from the literature.^[39]^ Relaxation superoperators were computed by *Spinach* using Redfield theory,^[40]^ as described in.[^31b^, ^41^] Rotational correlation times were fitted independently for the inside and outside of the erythrocytes. Translocation rate constants, which are distinct for α‐ and β‐anomers, were also treated as fitting parameters in the simulations.

Anisotropies of chemical shift tensors required by Redfield theory^[40]^ were estimated using the gauge‐independent atomic orbitals method^[42]^ running within DFT M06^[43]^ electronic structure theory model using cc‐pVTZ basis set^[44]^ in SMD water.^[45]^ Internuclear magnetic dipole interaction tensors were estimated from molecular geometries derived by minimising M06/cc‐pVTZ energies in the SMD water. The Gaussian16 package^[46]^ was used for all electronic structure theory calculations.

**NMR data fitting**. 2D EXSY spectra were fitted by numerical (Nelder‐Mead simplex) minimisation of the square of the Frobenius norm of the difference between the matrices representing the experimental and the simulated spectrum. In Table S1, *J*‐coupling signs (not constrained by the experiment) came from GIAO DFT M06/cc‐PVTZ estimates in SMD water. In view of the practical difficulty of obtaining and measuring a sufficient number of human blood samples for inter‐experiment statistics, standard deviations were estimated in each case from a single 2D EXSY spectrum by the bootstrapping procedure: 32 rounds of fitting were performed with half of the points in the experimental data randomly dropped from consideration.

## Supporting Information

Supporting Information (additional information on the statistical analysis) is available.

## Conflict of interest

All authors (D. S., C. Q. F., B. L., I. K. and P. W. K.) declare no conflicts of interest in the work, and the reporting of it, in this article.

1

## Supporting information

As a service to our authors and readers, this journal provides supporting information supplied by the authors. Such materials are peer reviewed and may be re‐organized for online delivery, but are not copy‐edited or typeset. Technical support issues arising from supporting information (other than missing files) should be addressed to the authors.

Supporting Information

## Data Availability

The data that support the findings of this study are available from the corresponding author upon reasonable request.
