# Supplementary material for: Quantitative Analysis of 2D EXSY NMR Spectra of Strongly Coupled Spin Systems in Transmembrane Exchange
Source: Chembiochem. 2023 Dec 4;25(3):e202300597. doi: 10.1002/cbic.202300597 (PMC10952724; doi:10.1002/cbic.202300597)
Supplement: Supplementary file 1 — Supporting Information [file CBIC-25-0-s001.pdf]

ChemBioChem

Supporting Information

Quantitative Analysis of 2D EXSY NMR Spectra of Strongly Coupled Spin Systems in Transmembrane Exchange

Dmitry Shishmarev,* Clement Q. Fontenelle, Bruno Linclau, Ilya Kuprov,* and Philip W. Kuchel*

Additional information on the statistical analysis (Figure S1), and the fitted parameters (variates) in the mathematical/physical model of the exchanging nuclear spin system (Table S1).

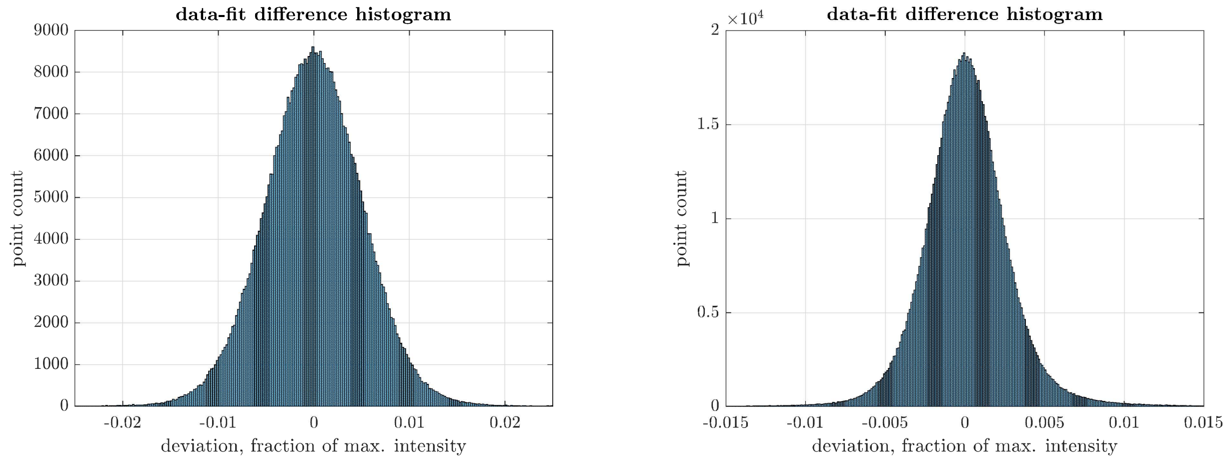

Figure S1. *Deviation histograms pertaining to the fitted simulations that are presented in Figure 4 of the main text.*

Table S1. ^{19}F 2D-EXSY parameters pertaining to the fitted simulations that are presented in Figure 3 of the main text.

<table>
<tr><th rowspan="2">Substance</th><th rowspan="2">Parameter</th><th colspan="2">α-anomer</th><th colspan="2">β-anomer</th></tr>
<tr><th>inside</th><th>outside</th><th>inside</th><th>outside</th></tr>
<tr><td rowspan="6">FDG33</td><td>Chemical shifts, ppm</td><td>$\delta_1 = -113.678 \pm 0.002$
$\delta_2 = -129.677 \pm 0.002$</td><td>$\delta_1 = -113.880 \pm 0.002$
$\delta_2 = -129.800 \pm 0.002$</td><td>$\delta_1 = -116.274 \pm 0.002$
$\delta_2 = -134.234 \pm 0.002$</td><td>$\delta_1 = -116.456 \pm 0.002$
$\delta_2 = -134.324 \pm 0.002$</td></tr>
<tr><td>J-coupling constant, Hz</td><td colspan="2">$J_{1,2} = 238.1 \pm 0.3$</td><td colspan="2">$J_{1,2} = 239.2 \pm 0.3$</td></tr>
<tr><td>Apparent exchange rate constant, s^{-1}</td><td colspan="2">$k_{oi} = 0.11 \pm 0.03$
$k_{io} = 0.23 \pm 0.03$</td><td colspan="2">$k_{oi} = 0.43 \pm 0.03$
$k_{io} = 0.79 \pm 0.03$</td></tr>
<tr><td>Equilibrium inside/outside ratio</td><td colspan="2">0.48 ± 0.19</td><td colspan="2">0.54 ± 0.06</td></tr>
<tr><td>Equilibrium α/β ratio</td><td colspan="4">0.27 ± 0.01</td></tr>
<tr><td>Rotational correlation time, seconds</td><td>$10^{-9.03 \pm 0.01}$</td><td>$10^{-9.28 \pm 0.01}$</td><td>$10^{-9.03 \pm 0.01}$</td><td>$10^{-9.28 \pm 0.01}$</td></tr>
<tr><td rowspan="3">FDG2233</td><td>Chemical shift, ppm</td><td>$\delta_1 = -120.538 \pm 0.005$
$\delta_2 = -133.943 \pm 0.005$
$\delta_3 = -129.317 \pm 0.005$
$\delta_4 = -129.532 \pm 0.005$</td><td>$\delta_1 = -120.836 \pm 0.005$
$\delta_2 = -134.276 \pm 0.005$
$\delta_3 = -129.599 \pm 0.005$
$\delta_4 = -129.753 \pm 0.005$</td><td>$\delta_1 = -136.464 \pm 0.005$
$\delta_2 = -139.534 \pm 0.005$
$\delta_3 = -131.854 \pm 0.005$
$\delta_4 = -132.120 \pm 0.005$</td><td>$\delta_1 = -136.779 \pm 0.005$
$\delta_2 = -139.698 \pm 0.005$
$\delta_3 = -132.290 \pm 0.005$
$\delta_4 = -132.192 \pm 0.005$</td></tr>
<tr><td>J-coupling constant, Hz</td><td colspan="2">$J_{1,2} = 271.3 \pm 0.4$, $J_{3,4} = 271.3 \pm 0.4$
$J_{1,3} = 0.5 \pm 0.4$, $J_{1,4} = -26.0 \pm 0.4$
$J_{2,3} = 10.0 \pm 0.4$, $J_{2,4} = -40.8 \pm 0.4$</td><td colspan="2">$J_{1,2} = 263.3 \pm 0.4$, $J_{3,4} = 263.3 \pm 0.4$
$J_{1,3} = 1.5 \pm 0.4$, $J_{1,4} = -28.5 \pm 0.4$
$J_{2,3} = 3.6 \pm 0.4$, $J_{2,4} = -23.0 \pm 0.4$</td></tr>
<tr><td>Apparent exchange rate constant, s^{-1}</td><td colspan="2">$k_{oi} = 1.67 \pm 0.12$
$k_{io} = 0.94 \pm 0.05$</td><td colspan="2">$k_{oi} = 1.37 \pm 0.07$
$k_{io} = 0.87 \pm 0.04$</td></tr>
</table>

	Equilibrium inside/outside ratio	1.78 ± 0.22		1.57 ± 0.15	
	Equilibrium α/β ratio	1.00 ± 0.04			
	Rotational correlation time, ns	$10^{-8.46 \pm 0.01}$	$10^{-9.09 \pm 0.02}$	$10^{-8.46 \pm 0.01}$	$10^{-9.09 \pm 0.02}$
